# Draft Genome Sequence of *Pantoea ananatis* Strain LMG 2665^T^, a Bacterial Pathogen of Pineapple Fruitlets

**DOI:** 10.1128/genomeA.00489-14

**Published:** 2014-05-22

**Authors:** Zaky Adam, James T. Tambong, Christopher T. Lewis, C. André Lévesque, Wen Chen, Eden S. P. Bromfield, Izhar U. H. Khan, Renlin Xu

**Affiliations:** Eastern Cereal and Oilseed Research Center (ECORC), Agriculture and Agri-Food Canada, Ottawa, Ontario, Canada

## Abstract

We report the draft genome sequence of *Pantoea ananatis* LMG 2665^T^, the bacterial causal agent of pineapple fruitlet rot.

## GENOME ANNOUNCEMENT

*Pantoea ananatis* is pathogenic on several economically important agricultural crops including pineapple (1), onions (2), maize (3), and rice (4). To date, five *P. ananatis* genomes have been published but none is the type strain. We report the draft genome sequence of the type strain of *P. ananatis* LMG 2665, the bacterial causal agent of the fruitlet rot of pineapple.

The draft genome of *P. ananatis* LMG 2665^T^ was determined by paired-end sequencing using an Illumina HiSeq 2500 with TrueSeq V3 chemistry at the National Research Council Canada (Saskatoon, Saskatchewan, Canada). A total of 18,809,898 pair-end reads, each 101 bp in length, totaling 1,899,799,698 bp, were obtained from 300-bp inserts. Quality checking using FastQC (http://www.bioinformatics.babraham.ac.uk/projects/fastqc/) showed that the reads are of sufficiently good quality such that no further trimming or error correction was required. Initial *de novo* assembly, using ABYSS v1.3.6 (5), produced 88 contigs contained in 73 scaffolds, from which scaffolds with length <300 bp were removed. The remaining 29 scaffolds (minimum, 1,050 bp; maximum, 612,849 bp; *N*_50_, 307,200 bp; total size, 4,980,044 bp; total number of unknown nucleotides [Ns], 201) were used for further analyses. SSPACE v2.0 (6) was applied on the resulting scaffolds to possibly extend and merge them into larger scaffolds based on read-pair information and short overlaps. This process reduced the number of scaffolds to 27 (minimum, 1050 bp; maximum, 612,849 bp; *N*_50_, 307,200 bp; total size, 4,980,628 bp; total number of Ns, 203). GapFiller v1.11 (7) was then used to close the gaps between the short scaffolds, that are contained within the large 27 scaffolds, by replacing the unknown nucleotides (Ns) with true nucleotides based on read-pair information and short overlaps. The final draft genome consists of 27 scaffolds (minimum, 1,050 bp; maximum, 612,855 bp; *N*_50_, 307,200 bp) totaling 4,980,528 bp with 50 Ns. The G+C content of the draft genome is 53.40% with an overall estimated coverage at 380×.

Mauve Contig Mover v2.3.1 (8) was applied to order the draft genome of the *P. ananatis* strain LMG 2665^T^ using *Pantoea ananatis* AJ13355 (accession no. NC_017531.1) as a reference genome. Automated annotation using the RAST annotation server (9) revealed that the draft genome of *P. ananatis* LMG 2665^T^ contains 4,787 predicted protein-coding sequences, of which 3,749 have assigned functions, 267 have proposed functions, and 771 have been considered hypothetical proteins, respectively. The draft genome also contains 88 predicted noncoding RNAs including 62 tRNAs and 16 rRNAs. The number of gene copies encoding 16S rRNA, 5S rRNA, and 23S rRNA are 4, 7, and 5, respectively. Compared to RAST, Glimmer v3.02 (10) using open reading frames (ORFs) as a training set predicted 4,893 genes, whereas RNAmmer v1.2 (11) predicted 14 rRNAs containing 1 copy of 16S rRNA, 8 copies of 5S rRNA, and 5 copies of 23S rRNA genes. However, tRNAscan-SE v1.3.1 (12) predicted 72 tRNAs genes.

### Nucleotide sequence accession numbers.

This whole-genome shotgun project has been deposited at DDBJ/EMBL/GenBank under the accession no. JFZU00000000. The version described in this paper is the first version, JFZU01000000.
